# Increased Tendon Thickness in Healthy Collegiate Baseball Pitchers Is Associated With Fewer Years of Pitching and Less External Rotation Motion Adjusted for Humeral Torsion

**DOI:** 10.1177/19417381261460866

**Published:** 2026-07-09

**Authors:** Matthew D. Heindel, Oscar Vila-Dieguez, Elizabeth Weiner, Daniel Awokuse, Nicholas J. Lobb, Lori A. Michener

**Affiliations:** † Division of Biokinesiology and Physical Therapy, University of Southern California, Los Angeles, California; ‡ Peak Performance Project, Santa Barbara, California

**Keywords:** humeral torsion, repetitive loading, shoulder, tendon structure

## Abstract

**Background:**

Mechanistic models of rotator cuff (RC) tendinopathy implicate repetitive loading as a driver of tendon structural changes. Baseball pitchers experience repetitive loading and physical adaptations in shoulder range of motion (ROM) and humeral torsion (HT) that may affect tendon structure and lead to tendinopathy.

**Purpose:**

To (1) characterize the effect of repetitive loading by determining dominant-to-nondominant arm differences in shoulder ROM, HT, and RC tendon thickness in healthy collegiate pitchers and (2) determine whether side-to-side differences in tendon thickness are associated with shoulder ROM, HT, and self-reported years of pitching experience.

**Study Design:**

Cross-sectional.

**Level of Evidence:**

Level 3.

**Methods:**

A total of 71 healthy, Division I pitchers were assessed bilaterally for shoulder internal rotation (IR), external rotation (ER), and flexion. HT and RC tendon thickness were measured via ultrasound. ROM measures were adjusted for HT to account for individual differences in bony morphology. Years of pitching was recorded. Paired *t* tests compared dominant vs nondominant arms (Aim 1). Multivariable linear regression, following LASSO selection, predicted side-to-side differences in tendon thickness using ER adjusted for HT and years pitching (Aim 2).

**Results:**

Aim 1: dominant arms showed greater tendon thickness and ER, and less IR, flexion, and HT (greater retrotorsion) (*P* < 0.01). Aim 2: greater tendon thickness was associated with fewer years pitching (–0.1 mm per year; *P* = 0.02) and had a limited, nonsignificant relationship with less ER ROM-adjusted for HT (*P* = 0.14) (model: adjusted *r*-squared = 0.08; *P* = 0.04).

**Conclusion:**

There are ROM, tendon structure, and HT adaptations in pitchers’ dominant arm. Greater tendon thickness was moderately associated with fewer years of pitching and was not significantly associated with less difference in ER ROM-adjusted for HT.

**Clinical Relevance:**

These findings are hypothesis-generating and support future longitudinal work incorporating exposure to pitching with bony morphology to understand their combined relationship with tendon morphology and potential injury risk.

Baseball pitchers undergo asymmetrical repetitive loading to the shoulders, which leads to adaptations in humeral rotational anatomy, soft tissue flexibility, and tendon structure.^3,10,18,19,24,34^ These adaptations to shoulder range of motion (ROM) and bony morphology of humeral torsion in the throwing arm are linked to shoulder injury risk.^3,10,18,19,24,34^ Insufficient shoulder external rotation (ER), internal rotation (IR), total rotational ROM, and shoulder flexion ROM have been shown to increase injury risk for pitchers.^3,10,24,34^ Pitchers also exhibit decreased anatomical torsion (increased humeral retrotorsion) of the distal humerus relative to the proximal humerus, leaving the distal humerus relatively externally rotated on the throwing arm compared with the nonthrowing arm.^26^ This shift in humeral torsion accounts for most of the shift in the rotational ROM and is considered a normal adaptation to throwing before the proximal humeral epiphysis is ossified.^6,26^ The degree of humeral torsion is considered inconsistently an injury risk factor in predictive models, limiting the understanding of the influence of humeral torsion and shoulder ROM adaptations on tendon health.^6,18,19,21,22,31,35^ If decreased ER is associated with shoulder injury risk and humeral torsion explains most of the variance in rotational ROM in pitchers, then humeral torsion could be an important factor to differences in shoulder tendon morphology and potential injury risk.

The rotator cuff (RC) tendons undergo remodeling from repetitive loading, similar to changes in bony morphology and ROM adaptations occurring in pitchers.^29^ However, the relationship between humeral torsion and tendon adaptations is unknown. The supraspinatus and infraspinatus tendons are common sites of RC injury in baseball pitchers, and increased tendon thickness is a common structural deficit associated with shoulder pain.^1,8,13,25,33^ Mechanistic models of painful RC tendinopathy indicate that tendon thickening is related to sustained repetitive shoulder loading,^25^ along with increased ER and decreased IR ROM. However, no study has controlled for person-specific factors of humeral torsion on ROM deficits by considering the nondominant side as a natural control.^6,9^

This study aimed to determine the effect of repetitive loading by characterizing (1) shoulder ROM, superior RC tendon thickness, and humeral torsion differences between the throwing and nonthrowing arms and (2) the difference in tendon thickness between the throwing and nonthrowing arms explained by differences in shoulder ER ROM, IR ROM, and humeral torsion. We hypothesize that tendon thickness will be greater in the throwing shoulder compared with the nonthrowing shoulder, and thickness will be related to shoulder ROM adjusted for humeral torsion and the number of years pitched. Defining the contributions of bony morphology, shoulder ROM, and exposure to pitching may help shoulder tendon morphology and inform future longitudinal work that will inform shoulder injury risk.

## Methods

### Participants

A total of 71 Division I collegiate baseball pitchers were recruited as a multisite project evaluating performance and risk factors for collegiate baseball players from July 2019 to February 2022. Demographics are described in Table 1. Inclusion criteria were age ≥18 years, no shoulder or elbow pain while throwing in the last 48 hours, no current elbow injury, and no history of any injury requiring ≥2 weeks of rest within the last 6 months from the time of testing. Pitchers who did not report ≥90% readiness to throw were excluded. All participants read and signed an informed consent form before participation. The study was approved by the University of Southern California’s Institutional Review Board.

**Table 1.** Descriptive statistics for n = 71 pitchers

Characteristic	Mean ± SD or n (%)
Age, years (range)	19.9 ± 1.3 (18-24)
Weight, kg	90.8 ± 7.3
Height, m	1.9 ± 0.05
Handedness, right; left	52 (73%); 19 (27%)
Pitching experience, years	8.9 ± 3.9
Race, n	White: 62 (87.3%) Asian: 2 (2.8%) Multiple races: 2 (2.8%) Other: 2 (2.8%) Not answered: 3 (4.3%)
Ethnicity, n	Non-Hispanic/White: 64 (90.1%) Hispanic/Latino: 3 (4.3%) Not answered: 2 (2.8%) Other: 2 (2.8%)

A questionnaire regarding participant injury history was collected via REDCap - a secure, HIPAA-compliant platform.^5^ The participant’s number of years of pitching was assessed via self-report. The number of years the participant has been pitching is of particular importance, as humeral torsion occurs primarily at the proximal epiphysis before bony maturation, as the forces of throwing are distributed disproportionately through the weaker cartilage than the surrounding stronger osseous structures.^12,27^ More time throwing before bony maturation could lead to greater bony adaptation. On the day of testing, the participant’s shoulder pain and soreness were assessed using a 0 to 10 visual analog scale (10 = worst pain and soreness). The dominant arm was recorded as the arm the participant throws with. Arm dominance was included based upon previous evidence showing that the ROM and humeral torsion relationship is different between those who are right- and left-handed.^32^ To control for participant-specific independent variables, a difference between the dominant arm and nondominant arm was calculated for tendon thickness and each ROM measurement.

No previous work has examined predictors of RC tendon thickness while controlling for the nondominant arm, so we conducted a pilot study of n = 20 collegiate pitchers to estimate the sample size needed to detect a significant effect of tendon thickness. The observed mean difference was 0.4 mm (7.0 mm vs 6.6 mm) with a pooled standard deviation of 0.8 mm, corresponding to a medium effect size of 0.5 (Cohen’s *D*). Based on these values, an a priori power analysis using G*Power (Version 3.1.9.7) indicated that a total sample of 60 participants would provide 80% power at an alpha level of 0.05.^4^

### Superior RC Tendon Thickness

**Figure 1.**
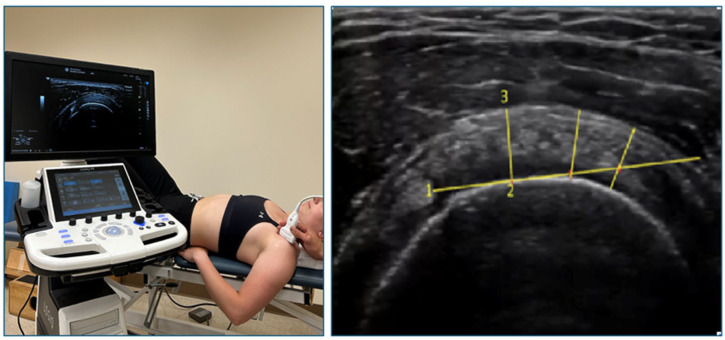
(Left) The participant is positioned in supine in the Crass position with their arm hanging off the edge of the table. (Right) B-mode image of cross-sectional supraspinatus and anterior infraspinatus tendon thickness. Tendon thickness was measured at 10 mm, 15 mm, and 20 mm from the lateral border of the long heads of the biceps tendon (1); 10 mm location is represented as the distance between 2 and 3. Lines are drawn at those points perpendicular to the orientation of the tendon fibers and tendon thickness was measured, and the average of the 3 represents tendon thickness.

Transverse ultrasound images of the supraspinatus and infraspinatus (Figure 1) were obtained bilaterally using a 6- to 12-MHz linear transducer ultrasound (P9; GE Healthcare). Two ultrasound images were obtained with the participant lying supine, with their palm placed on the ipsilateral iliac crest, with their elbow pointed inferiorly (supine Crass position). The transducer was placed perpendicular to the long axis of the supraspinatus and infraspinatus tendon on the anterior aspect of the shoulder, ensuring visualization of the long head of the biceps tendon as an anatomical reference. Tendon thickness was measured at 10 mm, 15 mm, and 20 mm lateral to the biceps tendon and averaged for a single measurement. The supraspinatus footprint extends 10 mm to 15 mm posterior to the bicipital groove, so the 15 mm to 20 mm measurements likely include the anterior portion of the infraspinatus tendon.^15^ Therefore, this measurement will be referred to as the ‘superior RC tendon thickness’ to reflect the potential contributions of both the supraspinatus and infraspinatus. Measurements on 2 images were averaged for data analysis. A side-to-side difference variable = throwing arm – nonthrowing arm, was created from the average measures. Test-retest reliability was established before data collection on 10 participants (intraclass correlation coefficient [ICC], 0.97; minimum detectable change [MDC], 0.4 mm), which is consistent with previous studies in our laboratory and others.^8,11^ In addition, this measure has been used to detect the onset of painful tendinopathy.^25^

### Humeral Torsion

**Figure 2.**
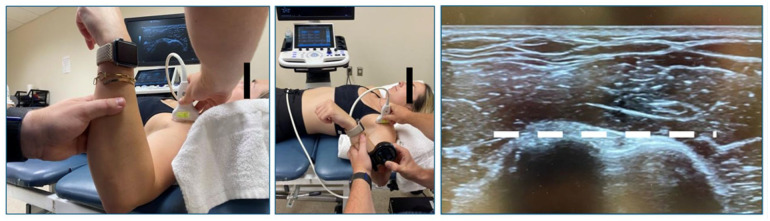
Measurement of humeral torsion. The humerus is initially positioned in 90° abduction in neutral rotation, as indicated by the yellow bubble inclinometer with the transducer visualizing the deepest part of the bicipital groove (left). The humerus is rotated internally (middle) until the greater and lesser tubercles are aligned in the horizontal plane (right). The angle for humeral torsion is then measured (middle).

Humeral torsion was measured bilaterally using a digital inclinometer, LogiQe ultrasound scanner with a 7.5 to 12.5 linear array transducer and bubble level. With the participant in supine and the arm abducted to 90° in the frontal plane, the transducer was placed cross-sectionally along the bicipital groove. The bicipital groove was scanned from the most distal to the most proximal portion to find the deepest point of the groove. The transducer was adjusted so the cortical bone was hyperechoic, and the bubble level was parallel to the horizontal plane (Figure 2, left). This position indicated the “0” position. Next, a second examiner internally rotated the shoulder (Figure 2, middle) until the greater and lesser tubercles were aligned with the horizontal plane (Figure 2, right). In this position, the degree of shoulder IR was measured by the angle of the forearm relative to the vertical using the inclinometer. Two measurements were taken and averaged for data analysis. A side-to-side difference variable = throwing arm – nonthrowing arm was created from the average measures. Test-retest reliability was established before data collection on 10 participants (ICC, 0.77; SEM, 1.3°; MDC, 3.0°) - an error magnitude similar to that reported by others.^2,16^

### Passive Shoulder ROM

**Figure 3.**
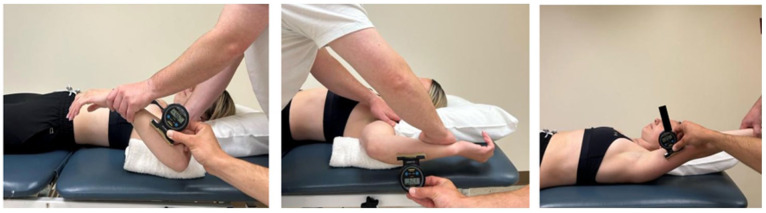
Measurements of shoulder IR (left), ER (middle), and flexion (right). ER, external rotation; IR, internal rotation.

Bilateral passive shoulder ROM was measured using a digital inclinometer. The inclinometer was calibrated to a vertical orientation for IR and ER and to a horizontal orientation for flexion, and the average of 2 trials was used for data analysis for each ROM measurement. The order of measurement of glenohumeral ROM was randomized. For glenohumeral IR and ER measurements, participants were positioned supine on the treatment table with their shoulder in 90° of abduction and the elbow in 90° of flexion. The humerus was supported by a towel roll with the elbow positioned slightly off the edge of the table. To measure clinical glenohumeral rotational ROM, the first examiner stabilized the humeral head and scapula against the table with one hand and passively moved the humerus into IR/ER with the other hand. The humerus was rotated until the first examiner detected the capsular end feel. Then, the second examiner placed the long axis of the inclinometer on the axis of the ulna and recorded the maximum IR and ER ROM (Figure 3, left and middle, respectively).

Glenohumeral total ROM (TROM) was calculated as the sum of the IR and ER ROM. Humeral torsion-adjusted IR ROM was calculated by subtracting humeral torsion from IR ROM, while ER ROM-adjusted for humeral torsion was calculated by adding humeral torsion to ER ROM. For the measure of shoulder flexion, the participant was positioned supine with their knees flexed to 90°. The first examiner placed one hand underneath the participant’s lumbar spine while grasping the distal humerus with the other hand. The first examiner flexed the participant’s shoulder until extension of the lumbar spine occurred. The second examiner then placed the long axis of the inclinometer along the axis of the humerus and recorded the value (Figure 3, right). Side-to-side differences were calculated by subtracting the nonthrowing arm from the throwing arm. Test-retest reliability was established before data collection on 10 participants; IR ROM (ICC, 0.95; SEM, 1.3°; MDC, 2.9°), ER ROM (ICC, 0.79; SEM, 3.6°; MDC, 8.3°), and flexion (ICC, 0.88; SEM, 2.6°; MDC, 5.9°).

### Statistical Analysis

Statistical analysis was performed with RStudio Version 2023.06.0 +421. Descriptive statistics are reported as means and standard deviations. Side-to-side differences (dominant – nondominant) were calculated and compared for each ROM of (ER, IR, TROM, flexion), humeral torsion, ER and IR adjusted for humeral torsion, and superior RC tendon thickness using paired samples *t* tests; alpha set at 0.05. For the model to predict side-to-side differences in tendon thickness, we applied a sparse multivariable linear regression using least absolute shrinkage and selection operator (LASSO) with the difference for the ROM and humeral torsion variables. In addition, years of pitching were included, as this has been associated with humeral torsion and injury risk.^7,27^ LASSO was used to select the predictors for the final model, due to the high dimensionality and multicollinearity of the predictors of superior RC tendon thickness. We determined the optimal regularization parameter (lambda) using 5-fold crossvalidation, and we performed 2000 bootstrap replications to ensure model stability and robustness. The final model was based on the lambda that minimized the crossvalidated mean squared error. To maximize interpretability, predictors demonstrating consistent selection across bootstrapped models were entered into a multivariable linear regression to predict differences in superior RC tendon thickness. Univariate Pearson correlations were performed to determine the individual relationships among variables retained in the final model.

## Results

Between arms, there was significantly greater ER ROM and tendon thickness, along with less IR ROM, flexion ROM, and humeral torsion (greater retrotorsion) in the dominant compared with the nondominant arm (Table 2). After feature selection, years pitching and ER ROM-adjusted for humeral torsion were selected as predictors of differences in tendon thickness. A multivariable linear model predicting differences in tendon thickness between sides was fit with differences between dominant and nondominant sides for ER ROM-adjusted for humeral torsion and years pitching as predictors with the dominant arm as a covariate of no interest.

**Table 2.** Descriptives for glenohumeral range of motion, humeral torsion, and superior RC tendon thickness, and mean differences and effect sizes comparing dominant with nondominant arms

Variable	Dominant shoulder	Nondominant shoulder	Mean difference [95% CI]	*P* value	Effect size
IR ROM, deg	46.3 ± 15.5	59.0 ± 14.8	–12.7 [–14.9, –10.5]	<0.01*	–1.4
ER ROM, deg	118.1 ± 12.5	106.8 ± 11.4	11.5 [9.0, 13.5]	<0.01*	1.2
TROM (internal + external), deg	164.4 ± 12.9	165.8 ± 13.6	–1.4 [–3.66, 0.8]	0.2	–0.2
Humeral torsion, deg	14.1 ± 10.7	26.2 ± 11.4	–12.1 [–14.4, –9.8]	<0.01*	–1.3
Flexion ROM, deg	173.6 ± 8.8	178.0 ± 9.6	–4.4 [–6.1, –2.7]	<0.01*	–0.6
IR ROM - adjusted for humeral torsion, deg: IR ROM - humeral torsion	32.2 ± 16.8	32.8 ± 16.2	–0.6 [–3.8, 2.7]	0.7	–0.04
ER ROM - adjusted for humeral torsion, deg: ER ROM + humeral torsion	132.1 ± 12.6	133.0 ± 12.6	–0.9 [–3.9,2.2]	0.61	–0.1
Superior RC tendon thickness, mm	7.5 ± 1.1	6.9 ± 1.0	0.6 [0.3,0.9]	<0.01*	0.5

Data are mean ± SD unless otherwise stated. ER, external rotation; IR, internal rotation; RC, rotator cuff; ROM, range of motion; TROM, total ROM.

* Significant difference between dominant and nondominant, paired samples *t* test (*P* < 0.05).

In the multivariable linear regression, years pitching was associated negatively with tendon thickness, with each additional year of pitching associated with a decrease of 0.08 mm in thickness (SE, 0.03; *t* = –2.4; *P* < 0.02). Similarly, ER ROM-adjusted for humeral torsion showed a negative association, where a 1-unit increase predicted a decrease of 0.02 mm in tendon thickness (SE, 0.01; *t* = –1.5; *P* = 0.14). The model explained 8% of the variance in tendon thickness differences (adjusted *R*-squared = 0.08; *P* = 0.04).

In univariate correlations, there was a negative relationship between years pitched and the difference in tendon thickness between sides (*r* = –0.21; *P* = 0.08). Years of pitching are also correlated negatively with the difference in humeral torsion between the dominant arm and nondominant arm (*r* = –0.15; *P* = 0.2). There is no difference in ER ROM based upon years of pitching (*r* = –0.05), but there is a greater bony adaptation in humeral torsion with more years of pitching (*r* = –0.15). Differences in ER ROM are related positively to differences in tendon thickness (*r* = 0.15).

## Discussion

Baseball pitchers load primarily the dominant arm, which allows for an ecological model to understand the effect of repetitive loading on the superior RC tendons, which is the most common site of shoulder tendon injury.^20^ Our findings indicate that (Aim 1) baseball pitchers experience alterations on their dominant shoulder in soft tissue structure (increased tendon thickness), bony architecture (decreased humeral torsion/increased retrotorsion), and shoulder ROM (decreased flexion and IR ROM, increased ER ROM) compared with their nondominant side. Notably, when IR and ER ROM are adjusted for humeral torsion, there is no longer a difference between the dominant and nondominant arms. This suggests that much of the ROM differences are due to the shift in humeral bony architecture, in agreement with previous literature.^17,26^ Our study was the first to show (Aim 2) a potential relationship between shoulder ROM, humeral torsion, and tendon thickness while controlling for person-specific factors by calculating the difference between sides. Fewer years of pitching and less difference in ER ROM-adjusted for humeral torsion between sides are associated with more tendon thickness, independent of arm dominance. In practical terms, pitchers who reported greater pitching experience had greater humeral retrotorsion on their throwing arm and less difference in tendon thickness between sides. This may indicate that force is distributed more through the tendon if pitchers lack humeral retrotorsion. However, joint loading and tendon stress were not measured, and these findings should be interpreted as associations rather than evidence of a specific protective mechanism.

A previous study examined the relationship between supraspinatus tendon thickness and dominant arm ROM in healthy baseball athletes.^9^ This previous study by Ishigaki et al^9^ showed 0.5 mm of greater supraspinatus tendon thickness on the pitching arm was very similar in magnitude (0.6 mm) to our study. However, there are some notable differences in methods. Their thickness measurement was in the longitudinal plane and sampled in only one location on the tendon. Our measurement was taken in the transverse plane and was an average of the tendon thickness across the tendon at 10 mm, 15 mm, and 20 mm. lateral to the long head of the biceps tendon. In addition, their sample included n = 22 collegiate baseball players, and only 4 of them were pitchers, while our sample included n = 71 healthy collegiate pitchers. Finally, we considered the nondominant arm as a control by calculating the differences between shoulders to characterize the relationships between physical adaptations and tendon structure, while Ishigaki et al^9^ used relationships only on the dominant arm.

Our findings indicate that repetitive shoulder loading is associated with tendon morphology, consistent with previous findings.^25^ The throwing arm had greater tendon thickness than the nonthrowing arm. In our multivariable model, dominant to nondominant tendon thickness differences were related modestly to the number of years pitching and ER ROM-adjusted for humeral torsion; however, the model only explained a small portion of variance (adjusted *r*-squared = 8%), and ER ROM-adjusted for humeral torsion was not statistically significant. Accordingly, these associations should be interpreted cautiously and should be used to generate hypotheses for future longitudinal studies. Univariate relationships showed a negative association between years pitched and side-to-side tendon thickness differences (*r* = –0.21; *P* = 0.08) and a negative association between years pitched and dominant-nondominant humeral torsion differences (*r* = –0.15; *P* = 0.2), suggesting that greater pitching experience may coincide with greater retrotorsion adaptation. Because the age of pitching onset was self-reported by the participant, and skeletal maturity was not measured directly, years pitched should be interpreted as cumulative experience rather than exposure only during skeletal immaturity. One plausible, but untested, interpretation is that interperson differences in bony morphology and ROM may relate to how tendon structure adapts to cumulative exposure to throwing. The limited variance explained also suggests that unmeasured contributors (e.g., pitch volume and intensity, pitch type/velocity, training load, scapular mechanics, strength, previous symptoms, and individual morphology/genetic factors) likely account for much of the observed variability. Finally, tendon stress, joint contact mechanics, and injury outcomes were not measured, so tendon thickness differences cannot be interpreted as protective, pathological, or predictive of internal impingement risk in this cohort.

### Limitations

There are limitations with this current study. First, we analyzed only participants who did not have shoulder or elbow pain in the last 48 hours and excluded pitchers with an injury over the last 6 months that required >2 weeks of rest. This limits inference as to why pitchers develop pain associated with RC tendon thickening and the factors that contribute to pathological thickening. For example, not all thickening may be pathological, and with ultrasound measures of RC thickness, we cannot be sure if the thickness is from hypertrophy or inflammation. The tendinopathy literature suggests that thickness is driven by an increase in water content from proteoglycan proliferation associated with inflammation.^28,30^ However, magnetic resonance imaging measures would detect more sensitively whether thickness is associated with inflammation versus hypertrophy. Second, this study is cross-sectional. Future prospective studies are needed to identify whether a lack of ER ROM-adjusted for humeral torsion predisposes a pitcher to develop painful RC tendinopathy and internal shoulder impingement. More specifically, longitudinal studies should explore the temporal relationship between change in bony morphology, change in tendon morphology, change in shoulder ROM, and injury risk. Third, several potentially important contributions to tendon morphology, including cumulative pitch volume and intensity, seasonal workload/training load, scapular and kinetic-chain mechanics, and shoulder strength, were not measured or controlled for. The participants were instructed not to work out their upper body for 24 hours before data collection, as this could change the tendon thickness.^14,23^ However, we cannot be sure that participants did not exercise their upper body.

## Conclusion

Repetitive loading adaptations on the dominant shoulder were found, including increased tendon thickness, decreased humeral torsion, decreased humeral flexion and IR ROM, and increased ER ROM in baseball pitchers. When rotational ROM was expressed relative to humeral torsion, dominant to nondominant differences in IR and ER were no longer observed, suggesting that bony morphology accounts for a substantial portion of rotational ROM differences. Side-to-side tendon thickness differences showed modest associations with years of pitching and torsion-adjusted ER; however, the multivariable model explained a small but significant proportion of the variance, and torsion-adjusted ER was not statistically significant. These findings are hypothesis-generating and do not establish mechanisms of protection or injury risk. Future longitudinal studies incorporating workload metrics and clinical outcomes are needed to clarify the clinical meaning of tendon thickness differences in pitchers.
